# Outsourcing Policy-Related Functions in Australia: Health and Equity Impacts

**DOI:** 10.1177/27551938251355452

**Published:** 2025-07-28

**Authors:** Julia Anaf, Toby Freeman, Fran Baum

**Affiliations:** 1 Stretton Health Equity, Stretton Institute, University of Adelaide, North Terrace Campus, Adelaide, Australia

**Keywords:** privatization, outsourcing, health, equity, democracy

## Abstract

Changes to the public sector in Australia over recent decades result from the adoption of neoliberal policies and New Public Management techniques. By the 1990s Australian governments were privatizing a significant portion of public sector roles, including outsourcing a range of traditional government services, policy, and decision making to the private sector, often to large global consultancy firms. While much is known about privatization and outsourcing, less is known about the health and equity impacts. Using a qualitative approach, data collection included documents, website searches, Parliament of Australia Hansard transcripts, media items, and semi-structured interviews (*n* = 11). Six key themes were identified, including the scope of outsourcing, consultants’ strategies, conflicts of interest, undermining the public sector, poor outcomes for the public, and implications for equity. The use of private sector actors in the Australian public sector has led to promoting private over public interests. There are legitimate reasons for governments to engage the services of global consulting firms in instances when public sector capacity cannot deliver specific highly specialized work. However, the current extensive use of consultants should be decreased through rebuilding public sector capacity to promote health and equity, and public over private interests.

## Background

Governments in many Organisation for Economic Co-operation and Development (OECD) member countries are now more likely to purchase than deliver services, regulate contracts rather than manage public servants, and focus on “managing an economy of incentives and opportunistic maneuvers than traditional forms of bureaucratic organization”.^1^ While initially concentrated in the United States and Westminster-based governments,^2^ this trend has been evident in Australia where changes to the public sector over recent decades have included extensive outsourcing of public services and policy functions.

By the 1990s, Australian governments were privatizing a significant portion of the public sector^3,4^ with approximately 80 privatizations documented between 1990–2014, including the banking, insurance, and telecommunications sectors, ports, airports, grain corporations, and many others.^5^ The Australian Hilmer Report^6^ culminated in the National Competition Policy to promote competition in public service provision, including through outsourcing.^7^ Privatization occurs via modes of financing, ownership, management, and product or service delivery.^8^ These divestitures were based on the ideological assumption that a shift from public to private ownership would yield efficiency gains.^9^ As a strategic instrument associated with globalization, privatization functions to expand the business–corporate sector; it creates “industrial reserve armies” by laying off workers and employees worldwide, removing their benefits and making them available for privatized businesses and corporations as cheap and temporary employees. This situation thus degrades working conditions.^10^ The role of the state is to focus more on maintaining social control over those “potentially explosive” sections of the population who are thus badly affected economically, politically, and socially.^10^ Privatization imposes an ideological culture of individualism and consumerism and creates new networks of political and governing elites who come from and are supported by business elites. They operate as agents of corporate capital by passing laws and regulations supporting privatization and globalization processes.^10^ Privatization also results in a loss of public assets and shrinks the public sphere.^10^ Often secure public sector jobs are replaced by insecure employment, and restructures once common only to the corporate world have also been embraced by public sectors. As well as privatization, there is also corporate capture of previous public sector activities and institutions, including universities.^11^

Whitfield provides a typology to help understand the pervasiveness of private sector reach. He defines four domains under which public services and the welfare state are transformed by privatization and marketization, including outsourcing.^12^ These domains relate to global public goods, privatization of assets and services, privatization of governance and democracy, and privatization of the public domain. The latter includes the primacy of market values, and privatization of public intellectual capital and public space.^12^ Whitfield's typology signifies the range and scope of mediating actions or recommendations necessary to deal with negative health and equity consequences of outsourcing.

Privatization involves outsourcing a range of traditional government services, policy, and decision-making roles to the private sector, many to large global consultancy firms. Concern has been growing about the power, influence, and threats to health, equity, and democracy by the operations of these firms.^13–15^ Questions have been raised about their integrity and their management of conflicts of interest (COIs),^14^ and have led to several Australian state and federal parliamentary inquiries.^15–19^ Global consultancy firms have been vital to the development of neoliberalism, and they have both promulgated it and benefited from it. This is seen as their roles and services have perpetuated privileging the private sector and neoliberal approaches to governance, and this widespread dependence on the private sector has created demand for their services.

White and colleagues^20^ state that these firms’ influence has grown due to the impact of globalization and increased economic uncertainty that creates a market for specialized advice. They promote themselves as analyzers of unique knowledge, which allows them access to “big data” that they suggest the public sector is unable to understand and synthesize. Due to global complexity and interconnectedness, and as governments worldwide downsize and outsource their public sector expertise, these firms promote the idea of certainty in a growing age of global uncertainty.^20^ Clearly linked to outsourcing public services and negative health and equity outcomes, Australia and other global jurisdictions engaged large global consultancy firms that received multi-million-dollar contracts during the COVID-19 pandemic response without clear transparency and accountability.^21^

Therefore, based on their capacity to influence governments globally, and in ways that affect public health and the public interest, global consultancy firms are an important commercial determinant of health (CDoH). They are part of “the systems, practices and pathways through which commercial actors drive health and equity”.^22,23^ In 2023, it was estimated that the global management consultancy market was worth over US$860 billion.^24^ Australia's consulting industry is the world's fourth largest, with spending per capita approximately double that of comparable countries such as Canada or Sweden.^25^ This is partly to deal with the impact of arbitrary public service staffing caps implemented in 2015 when the Australian Government announced that it would freeze public sector staffing at 2006–2007 levels, while concurrently funding a private “shadow” workforce consisting of staff from consultancy firms.^26^ An audit of government employment revealed that during the 2021–2022 financial year, approximately 54,000 full-time staff were employed as consultants, service providers, or labor hire while 144,300 staff were directly employed.^27,28^ This highlights the tension between the profit-driven nature of commercial actors and the public sector working to promote the public interest.^29^

A broad literature exists on privatization and outsourcing by Australian governments,^30–32^ but little is known about the actual and potential health and equity impacts from outsourcing policy-related functions and decision making, a gap this research seeks to fill. The focus is explicitly on the wider social and commercial determinants of health that have the greatest impact on population health outcomes. Our article examines the extent of the outsourcing of policy-related functions in the Australian national government, and the impacts of this outsourcing on health and equity. While these impacts are in one country, the global reach of the firms means the impacts are likely global.

## Methods

Data were collected from documents, website searches, Parliament of Australia Hansard transcripts, media items, and semi-structured interviews with respondents with public sector or private consultancy backgrounds (*n* = 11).

### Documents and Websites

The Australian Government Departments and Agencies website was checked to identify the scope of government departments which largely use private consultancy firms. The AusTender website was accessed for documents on the number and value of contracts awarded to each of the “Big Four” accounting/professional services firms (PWC, Deloitte, EY, KPMG) and the “Big Three” management and strategy firms (McKinsey, Boston Consulting, Bain and Co.) listed between 2005 and 2023.

Each of the Big Four and Big Three websites were searched for corporate data relating to government consulting roles, corporate social responsibility (CSR) strategies and partnerships, financial reports, audit, and transparency reports. We accessed the annual Australian Securities and Investment Commission (ASIC) reports evaluating each firm's operations. The Federal Senate Standing Committees on Finance and Public Administration were searched between 1999–2022 for final reports relevant to informing aspects of outsourcing government functions.

### Parliament of Australia Hansard Transcripts

Data included transcripts of public submissions to the 2023 Australian senate “Inquiry into Management and Assurance of Integrity by Consulting Services.” Key terms of reference included the management of risks to public sector integrity arising from the engagement of consultants, COIs, and issues of transparency and accountability affecting the public good.^15^

### Media Items

The NewsBank database, which consolidates current and archived information from newspaper titles and other media, was searched for items in the *Australian Financial Review* and *Canberra Times* between 2000–2023 using the search term “Australian government outsourcing.” These mainstream media outlets report widely on Australian government and business matters. The website of Michael West Media, an independent investigative online news service with strong reporting of the role and impact of global consulting firms, was searched from 2018 (inception) to 2023. The website of *The Guardian* news service was searched using the search term “outsourcing,” a more explicit term for the use of consultants than “privatization,” to identify any items discussing outsourcing policy-related advice to large consulting firms. Relevant items were identified in The Conversation, a network of not-for-profit media outlets publishing news stories and research reports online, and from the online publication *Pearls and Irritations*, an Australian online blog and platform for the exchange of ideas from a progressive, liberal perspective.

### Semi-Structured Interviews

Interview participants were selected for their ability to provide perspectives on outsourcing from people with public sector or academic backgrounds and those with consultancy or private sector backgrounds. Potential participants were identified by strategic internet searches, purposive and snowball sampling, and the social media platforms LinkedIn and Twitter (now X). Interview schedules were tailored to each interview cohort. Questions were designed to elicit responses concerning positive and negative impacts from outsourcing aspects of government policy-making and related functions in Australia.

All potential participants were emailed a personalized invitation, a participant information sheet, and consent form. Follow-up emails were sent if no reply was received after 14 days. Of the 10 participants approached with consulting backgrounds, six agreed to an interview and four did not respond. Of the 10 public sector actors approached, five agreed, one declined, and four did not respond. The 11 interviews were conducted by telephone or Zoom between October 2023 and March 2024 and were transcribed by professional transcription services.

One hundred and three items inclusive of documents, items from website searches, Hansard transcripts, and interview transcripts were imported into NVivo qualitative data software for thematic analysis. An a priori coding frame was developed by the coauthors and augmented by subcodes reflecting the research questions. Insights from the coding were discussed by the coauthors during team meetings to help identify key themes. Ethics approval to conduct the research was obtained from the University of Adelaide Human Research Ethics Committee (Approval Number H-2023-099, May 2023).

## Results

**Figure 1.**
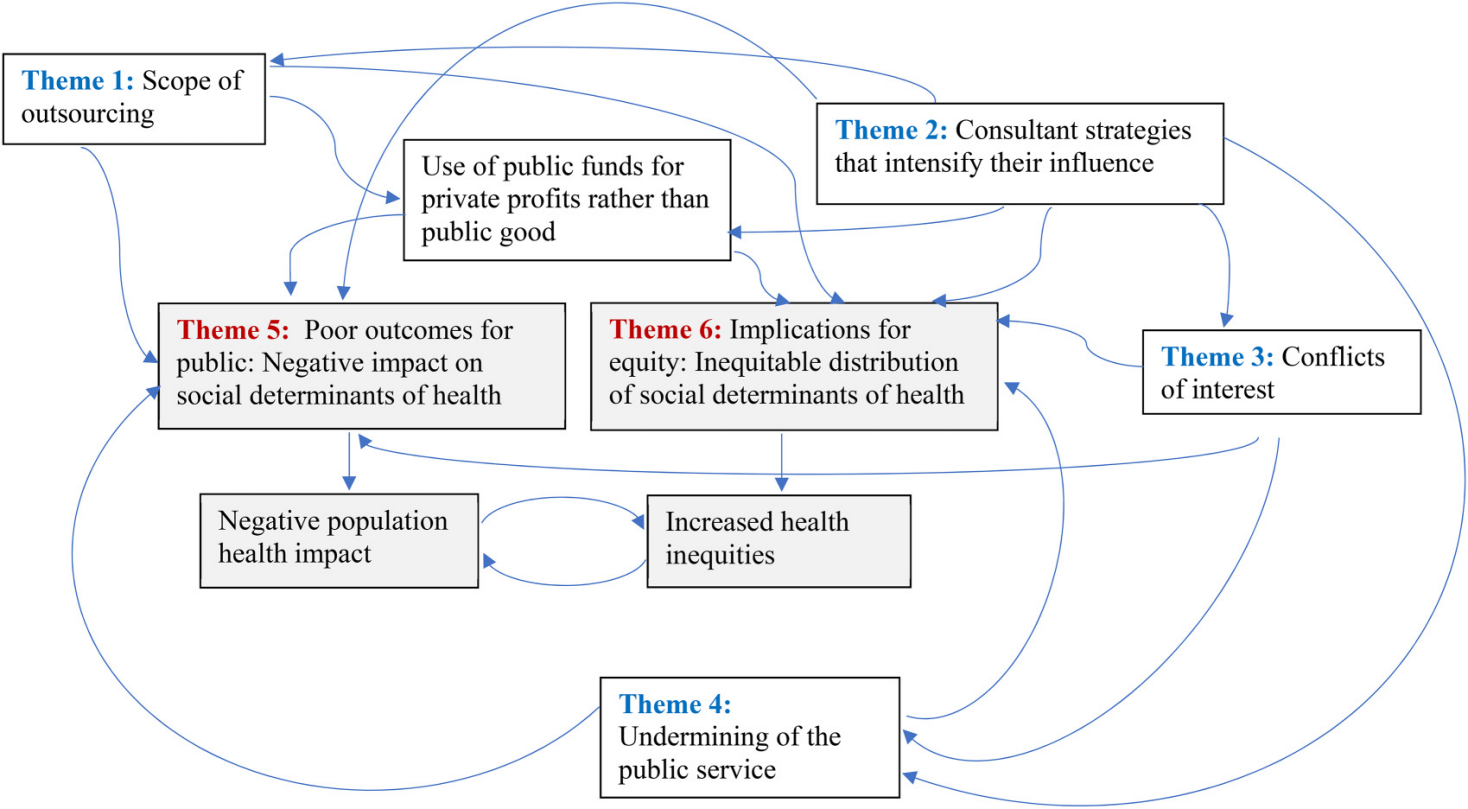
Interactions between consultancy companies’ practices and population health and equity outcomes.

We identified four themes that relate to the consultant firms’ mode of practice and interaction with the public sector: 1) scope of outsourcing, 2) consultant strategies, 3) COI, and 4) undermining the public sector. The last two themes relate to the outcomes of these practices and relationships: 5) poor outcomes for the public and 6) implications for equity. The complexity of the links between these themes is shown in Figure 1, which demonstrates the ways in which each impacts the others.

### Theme 1: Scope of Outsourcing

The scope of outsourcing government roles encompasses both industry sector reach and the level of public financial outlay to private interests.

*Industry Sector Reach*. Outsourcing has become embedded in the Australian Public Service (APS), with staff who are engaged as consultants, contractors, or through labor hire firms. Our findings revealed broad sector reach by private consultancies, with the highest value contracts being awarded between 2012–2013 to 2021–2022 by the Department of Defence, Department of Home Affairs, Services Australia, Department of Health and Aged Care, Department of Employment and Workplace Relations, and the Australian Taxation Office.^33^

Although the data revealed a largely critical focus on the nexus between outsourcing government policy-related functions and the public interest, it was generally agreed that there are legitimate reasons to engage private consultants to undertake public policy-related work in clearly defined circumstances. The scope of reach also extends to the tertiary education sector, with global consulting firms engaged in many aspects of university management and governance.^34^

*Financial Outlay*. We also found that over the past decade the total volume of consultancy work undertaken for the APS increased by 400 percent from AU$282 million in 2012–2013 to over AU$1.4 billion in 2021–2022.^24^ Although numerous consultancies are employed by government, the Big Four firms were the largest recipients by value of consultancy-related contracts, including tens of millions of dollars spent on global consulting firms. These firms included McKinsey and PwC for the COVID-19 vaccine rollout, without providing detailed evidence about the work they were undertaking. McKinsey's Australian arm was awarded AU$1.6 million to provide support services for the vaccine rollout over one month at a cost of almost AU$57,000 per day.^35^ The government also engaged McKinsey on an AU$6 million contract for climate modeling that was well within the capacity of the CSIRO to undertake.^36^ Insights into the reasons for high financial outlays came from one respondent from a large global consultancy firm:

I’d do all the proposal and the costings, etc., to submit and then I’d pass it to the partner whom I was working for, and he’d say, *“*You want to round that up? We can’t do it for that.” I’d say to him, *“*Hang on, this is ridiculous. No-one in [stated city] is going to pay that for me . . . . This is dreaming . . . .” So it's almost an imperative that comes from the Big Four that they have to charge out, in order to make any money.

### Theme 2: Consultant Strategies

The extent of outsourcing government roles is facilitated by the range of strategies. One is “land and expand” whereby recommendations to government are made with an aim to generate further work rather than to support the public interest.^37^ As one media commentator noted:

They don’t come in just to provide internal advice, they come in to provide advice on government policy. They tell you to privatise or to put in place commercial models, and then you need them to enact those changes.^38^

Another strategy is “lowballing” whereby consulting firms undercharge an initial contract to then be well placed to bid for related work.^39^ “Walking both sides of the street” is a strategy by which to derive income based on developing government policy while also being mindful of its impact on private clients. One aspect is obtaining confidential information from government clients that may benefit current or future private clients.^36^ An example we found was of consultancy firms being paid lucrative government contracts to audit the quality and safety of residential aged care homes while simultaneously charging aged care providers for advice concerning audits and accreditation.^40^

Lobbying governments and providing political donations are other key strategies.^41^ We found that the Big Four firms are the largest beneficiaries of taxpayer funding while also consistently among the largest donors to Australian political parties.^42^ Donations to the two major Australian political parties over the past decade totaled over AU$4 million.^36^ A respondent highlighted the consequences of gaining unequal access to decision makers:

I think one of the key problems is that the consultants are much better connected to the politicians than the bureaucrats, because they are amongst the biggest political donors in the country. They hang out with the politicians all the time. They have partners whose whole job it is just to go and hang out with them because they fund 50% of their revenue every year. It comes from government consulting or thereabouts. The master–servant thing breaks down a little bit in that context. (Former Big Four consultant)

The strategy of revolving doors describes staff movements between the public and private sectors. This may reduce accountability by diminishing institutional knowledge and memory in the public sector. One senate submission noted that this can also fail the public interest test due to the revolving door from the public sector to higher-paid outsourced roles, leading to an increase in wages, particularly for senior executives and chief executive officers (CEOs).^43^

Another potential risk is where consultancy firms are able to use insider knowledge from their staff who have been seconded to public service work or had access to inside information, and use that information in their work with other clients.^37^

### Theme 3: Conflicts of Interest

The above consultancy strategies involve COIs for both governments and consulting firms which arguably exist in a codependent relationship. These consultancies provide professional accountancy, business, and tax compliance services, as well as management and efficiency or profitability advice. The media analysis noted that this diverse range of services, which is claimed to be provided in an independent and ethical manner, is “well hidden behind commercial-in-confidence and strict secrecy contracts”.^44^ One senate submission highlighted negative impacts from the Australian Government's overdependence on consultants:

This dependence is corrosive to Australian democracy in two ways: it hollows out the capabilities and skills of the public service and it leads the government to make decisions based on advice that can be poor, compromised, or self-interested.^39^

Consultants gain extensive influence over health policy due to recurring government contracts. Under a neoliberal policy agenda this places profits above the public interest and risks creating negative health outcomes.^45^ The Public Service Act (1999) underpins the notion of the public sector serving the interests of government, the parliament, and the Australian public. This is in distinction to commercial entities supporting private vested interests.^46^ Interdepartmental cooperation is the distinguishing operational feature of the public service in comparison with the private sector.^46^ Therefore, there are far-reaching implications for global health and equity due to consulting firms, especially when they have offices across many jurisdictions. One respondent who worked for a large consulting firm in many different countries felt conflicted by being unable to maintain an ethical professional service due to pressure from their employer, a Big Four firm. Regardless of where they were employed, there was always the same demand to not expose government wrongdoing. The context of these conflicted feelings is expressed in the following exchange with a manager:

I’ve been working now with you for [x] years. We’ve discovered fraud after fraud after fraud. Clear evidence. In the beginning I thought, okay, I was not giving enough evidence. But now, how can you still deny this, and we still not report this? And he said, “Well, [named consultant], welcome. This is [named country] for you. This is how things work here . . . . If we expose and accuse the government of being corrupt, they will not hire us anywhere anymore.” (Former Big Four consultant)

The credibility of firms who claim to offer value for money to both their private and government clients is also challenged by our research. One respondent explained that as firms win only a small percentage of contracts:

There's an awful lot of work they’ve got to put in to win a contract and they’ve got to pull back that money somehow, and that money's going to come out of the contracts that they win. So, they’re going to be paying less and possibly doing less to do that. I think that is a big conflict of interest. (Unaffiliated private consultant)

Public servants are legally required to provide advice to government that is open, honest, timely, and based on the best available evidence. Being subject to the Freedom of Information law, their work provides both an incentive and obligation to comply with the Australian Public Service Act.^39^ However, the waning of such “frank and fearless” independent advice over recent decades, to suit conflicted mutual interests of both private providers and government, was highlighted by a respondent:

I think when things are badged as independent, or seen as independent, in a lot of cases they’re not, unless it's a Royal Commission. So I think that shocked me, and more so I saw it in [named firm], where a partner would be like, “No, get rid of that recommendation. I just spoke to [high ranking bureaucrat] and we can’t have that in there,” and that's a way to make sure that they get more work, because they’re not rocking the boat. (Former Big Four consultant)

A senate submission contextualized this situation by explaining that consultants are sometimes hired expressly to tell the government only what it wants to hear. This leads to a charge of misuse of public money in ways that help governments mislead the public about the merits of particular policies or plans.^39^ One consultant gave a more nuanced perspective on COIs and the use of public money:

I think they [consultants] can give value for money, because often, they can come in and they can be a sort of scapegoat. They can come in and make the hard decisions and people can blame it on them . . . . It just needs a fresh pair of eyes coming in to look at it and someone who's willing to ask the difficult questions. So, they can get value for money, but I think that comes down to what you’re paying them. My sense is these companies get paid too much. (Unaffiliated private consultant)

### Theme 4: Undermining the Public Sector

The work of the APS is grounded in the Westminster system that articulates the Australian Parliament's expectations of public servants to be impartial, apolitical, committed to service, open and accountable to the Australian community, respectful, and ethical.^47^ The scope and reach of outsourcing, the range of consultant strategies, and COIs all lead to undermining the APS due to both the financial constraints and the ideological imposts associated with outsourcing.

*Financial Constraints*. Interview respondents provided a particularly rich source of data on the financial constraints associated with outsourcing. One former public sector actor highlighted wasteful transaction costs associated with hiring consultants under a marketized procurement model. Another told of the initial cuts to middle-level management in the APS that ultimately led to higher costs and thus financial constraints on the public purse, stating:

So, they cut the guts out of the middle [management] and at that stage the opportunity arose, I think, for senior managers to decamp themselves into the consulting companies. But they took redundancies, large redundancies, and they went to work in the private sector, and then consulted back doing the same job they had been doing when they were public servants, for a lot more money. (Former public sector consultant)

Reducing the size of the public service also leads to a loss of institutional knowledge, thus:

You’ve got people there that aren’t able to answer the questions or come up with ideas, because they don’t have the experience or the understanding. And those people that do are now just charging themselves out for $3,000 a day to come back in and provide that advice. (Former consultant in both private and public sectors)

An interview respondent who had been a health care consultant and former public servant stated that there are consequences for workers in the health sector with implications from this loss of expertise. They noted, “We don’t hold ourselves accountable to outcomes and we wouldn’t know how to achieve an outcome if it smacked us in the face, frankly.”

The same respondent also noted other problems in relation to the use of private consultants by the federal Department of Health in relation to the decentralization of its primary health planning. As Whitfield's typology highlights, privatization leads to a loss of intellectual capital^12^ and:

Not having a centralised or core team of experts within the department that are able to provide advice and make decisions, it means, I think, that there's a lot of fragmentation. Health needs of populations are not even done by the Department of Health anymore, it's given down to the primary health networks, and they contract it out to consultants. So how that is then meant to help the Department make decisions, prioritise where there is need and all the things that they’re supposed to do? It just causes more problems than it solves because of the use of consultants. And they are core skills that I feel like a department/primary health network should be able to do; you know, analyse data, consult with community, understand what the need is.

#### Ideological Imposts

Neoliberal policies invoke individuals to take personal responsibility for their actions and circumstances.^48^ Yet these commentators argued that in practice this rarely happens, and public sectors are weakened as a result:

Political leaders in a democracy ought to be the very ones bearing *“*personal responsibility,” not those whose lives depend on their decisions, their management. Rarely are any of them held to account for the gutting of government, for the hundreds of millions of fees which go each year to consulting firms, for the corporate handouts.^49^

A submission to the senate inquiry also highlighted the negative impacts from governments adopting neoliberal ideology:

Nowhere is that ideology more prevalent than among accounting, finance, and management consultancies. It would be no exaggeration to attribute that ideology with having brought about all the chronic and self-perpetuating failures we have seen in education, social welfare, health, and aged care policies [that are] informed by its toxic “logic”.^34^

This view reflects the impacts from “hollowing out” the APS, whereby it is argued that the capacity of the nation-state is now merely a “shadow of what it was in the mid-twentieth century”.^50^ One former public sector actor argued that neoliberal ideological standpoints lead to increased costs, which in turn lead to reduced capacity for the public sector to work in the public interest. There is a lack of will to use public sector capability, with three interrelated reasons being a desire for secrecy, risk-aversion, and “rubber-stamping”.^51^ By contracting consultancy firms, governments can shield advice from scrutiny. The inclination towards secrecy reflects high-level risk aversion within both the government and the APS, and consultants can provide “cover” by rubber-stamping pre-determined government decisions.^51^ As Whitfield explains, undermining the public sector culminates in public assets instead being deployed to promote private interests, with poor outcomes for the public.^12^

### Theme 5: Poor Outcomes for the Public

A range of benefits to the private sector, including increased government funding and access for consultants that comes from outsourced government roles, is promoted by a regulatory environment reflecting a lack of scrutiny, transparency, and accountability. We found claims of a lack of transparency by the Australian Government in failing to publish key contracts on the AusTender website, arguing that information was “commercial-in-confidence”.^52^ More than 80 percent of the richest 200 Australians have made their fortunes in property, finance, mining, banking, and superannuation.^53^ A submission to the senate inquiry noted that these sectors are heavily regulated, and in which fortunes are made through favorable planning, legal, and regulatory exemptions. They include tax concessions and subsidies won for them by the Big Four and other consultancies influencing governments.^34^

Several senate inquiry submissions identified ways in which a lack of government scrutiny supports private interests. One argued that:

The joint provision of advisory, accounting and legal services by these firms can also provide a *“*cover” for legal privilege of documents and information, circumventing investigations into the myriad tax avoidance schemes that have been employed by multi-national companies, and to which I believe the Big Four are complicit (Mesch, 2023).

Another submission pointed to an environment of “soft corruption,” argued to take place through a “game of mates” or the social connections between people working in, between, and for departments, agencies, and other semi-autonomous bodies as well as within political parties, peak bodies, public relations firms, corporations, governments, and consultancy firms.^34^

A clear tension between commercial interests and public health is highlighted in what was revealed as a major government scandal involving a private consultancy firm—in 2022 a Royal Commission examined the “Robodebt” program. Between 2015 and 2019 the Australian Government unlawfully accused hundreds of thousands of vulnerable welfare recipients of owing money. This program, now deemed illegal, calculated debts relying on flawed algorithms employed without human oversight. The Royal Commission found that this plan was “crude and cruel, made victims feel like criminals and dole cheats, and the despair that it created caused negative health impacts including suicides”.^54^

One Big Four consultancy firm, PwC, was implicated in the scandal, with a senate inquiry submission subsequently documenting how government advice from global consultancy firms can be procured but not delivered, or delivered but not acted upon.^37^ It raised the public perception that governments use private consultants to provide preferred advice, while circumventing the accountability and transparency demanded of departmental advice. This submission noted that, unlike advice received from consultants, departmental advice is readily accessible by Freedom of Information provisions, is subject to Senate Estimates and other parliamentary oversight, and demands ministerial accountability.^37^

Another senate inquiry submission explained how the Robodebt case indicates a lack of transparency and accountability by noting that a short PowerPoint presentation by PwC became the agreed deliverable by government for a million-dollar consulting outlay. This was claimed to significantly undermine public confidence in government and public sector integrity.^55^ Unlike other professional services including auditing and law, management consulting is not a protected or regulated profession. Despite attempts to introduce governing standards and norms, there remains no international binding standard.^56^

One respondent who worked as a consultant in both public and private sectors argued that private consultants gained both disproportionate and negative influence over government, and should only be allowed to provide limited input into decision making:

[The government] shouldn’t be outsourcing the whole thing and getting the consulting firm to do the whole thing, the analysis, the review, the consultations, the writing, the whole thing. I think the department should hold the pen . . . . I see them [private consultancies] as negatives for society. There are so many core skills that are now outsourced, not just policy-making ones.

A senate inquiry submission by a former Big Four consultant highlighted unaddressed ethics scandals and a “sustained decline in audit quality.” It maintained that professional standards are falling because self-regulation is failing, arguing that “Australia is unusual in our continuing reliance on self-regulation of the accounting profession”.^57^ An argument was raised that the public has a right to ask why public money is going to shareholders of private companies as profits, dividends, or high executive salaries when it could otherwise be retained in government to pay for public services.^58^

### Theme 6: Implications for Equity

We found a range of implications for equity from the shifting focus from public to private interests:

A predisposition by recent governments to outsource human services risks poorer outcomes for the most disadvantaged and erodes public sector capability to design and (where necessary) deliver effective services for the most vulnerable.^59^

Several respondents highlighted that implications for equity arise from the use of private consultancies that do not prioritize equity:

Often, particularly in the public health sector, we’re dealing with issues around working with marginalised groups, low socioeconomic groups . . . so it should not be based necessarily on profit. I don’t think necessarily for these consultancy companies that it is a priority for them. There should be some sort of capping and transparency and more diversity in the consultancy sector. (Unaffiliated private consultant)

. . . I think the big problem that I see is that whilst private sector consultants are typically very well trained in matters of managerialist fashions and sort of the scramble for economic efficiency, very little policy advice that I see talks about the age-old creeping issue of equity and equitable treatment of the winners and losers. (Former public sector actor and academic)

Another respondent focused on the lack of equity across the wide scope of government services in a hollowed-out public service, resulting in differential impacts on:

. . . the poor and the strugglers, whether it's getting access to early childhood, getting a job, getting into a decent residential aged care facility, getting access to community health services, getting into a public hospital if you need it, rather than having to pay a lot of money in the private sector. The consequences of this outsourcing are dire for the bottom 40% or 50% of the population, in my view. (Former public sector actor)

A private consultant who worked globally spoke of the need for, and difficulties in gaining, highly skilled people to work in ways to promote health and equity among groups in vulnerable circumstances. This situation indicated the need for more than a transactional or profit-driven approach for dealing with:

. . . the marginalised groups, the vulnerable groups . . . . Because to reach these people, to develop policies or to work with these groups, whether they’re newly arrived migrants with different languages, different cultures, people with disabilities, homeless people with cancer, Indigenous peoples living in isolated communities or wherever, in order to work with these people, you have to employ people with very specific skills that can work with them . . . and these people are in short supply. Consultancy companies in my experience don’t see these people as an asset. They want to go in with an expert who's going to come in and do the job. (Unaffiliated private consultant)

### Addressing Health and Equity Impacts

The data also included numerous recommendations for dealing with the negative impacts for health and equity from outsourcing to global consultancy firms. While not all can be recounted, many were highlighted in the submissions to the Parliamentary Inquiry. Those recurring and therefore key recommendations are discussed here.

One recommendation is to restore public sector capacity. This is arguably indicative of the critical role of the public sector in supporting health and equity. This can be achieved through embedding training and capacity-building for public servants into consultancy contracts to recognize that governments act as economic “value-creators” instead of being negatively cast as a “wasteful and inefficient value extractors”.^39,55,56^ Another way is to assist in rebuilding the APS by establishing a statutory body that adopts a whole of government approach to procuring consulting services. This body would report regularly to provide transparency, make recommendations, and employ qualified public servants to oversee contracts and consulting activities. This requires investing in creating internal capacity and capability by ensuring that the public sector attracts career-oriented “competent, purpose-oriented and curious individuals”.^34^ Consultants and contractors should be mandated to include the transfer of their skills and knowledge to APS staff as part of their contracts, thereby providing savings from reducing the ongoing need for consultants and instead reinvesting in APS staff.^60^

Another key recommendation is to abolish caps on public sector recruitment as was called for in the 2019 independent review of the APS^61^ and the 2020 senate inquiry, which examined changes to service delivery models on the administration and running of government programs.^62^ Abolishing staffing caps would bolster internal capacity instead of using expensive external consultants.^39^

There are recommendations to limit the use of commercial-in-confidence provisions in government contracts by requiring tenders to prohibit their inclusion without unequivocal independent advice that such measures are necessary.^58^ We also found wide-ranging recommendations for a structural split within major global consultancy firms between audit and non-audit functions.^51,63,64^ COIs between these two roles lead to a lack of auditor independence. This problem can have wide-reaching implications for institutional accountability and financial stability. Accurate auditing is a critical factor for economic stability in global markets with potential for broader social and economic impacts from audit failures, such as those that occurred in the global financial crisis (GFC) in 2008–2009.^65^ Sikka^66^ detailed evidence that many positive audits were shown to be inaccurate for firms that failed in the crisis. They note that these events raise questions about the value of company audits, auditor independence, and quality of audit work, economic incentives for good audits, and the knowledge base of auditors. Inaccurate audits had real-world impacts in the GFC, which affected tens of millions of people across different nation states and may have been avoided had companies been accurately audited and their shortcomings found earlier.

Another recommendation is to deal with the ramifications of revolving door strategies, where more detailed and strict policy is required, potentially including a mandatory post-separation period for public servants who have dealings with consulting firms.^39^ A further recommendation is to restrict political donations from private firms that are eligible for government contracts.^58^ Another is to implement a national debarment regime that would prohibit consultants who have committed serious misconduct or have been convicted of a criminal offence from providing services to the government.

Extending the Freedom of Information Act to include private sector bodies that are contracted to provide services or functions to the public on behalf of the government is a further recommendation. This would allow for Freedom of Information requests to be utilized to obtain relevant documents.^58^ There have also been calls for far greater “whistleblower” protections for public servants that are essential for revealing unethical behavior, corruption, and COIs.^60^

## Discussion

There is not one single solution to eradicating negative impacts from harmful business models, practices, and products of private actors. Addressing the commercial determinants of health and health inequities demands rebalancing power asymmetries.^67^ The past four decades of neo-liberal policies have seen a change of governments’ arrangements from government to governance, from direct state management to steering at a distance—“steering not rowing”.^68,69^ Government actors must instead harness their structural power to challenge commercial actors, including global consultancy firms, who both work to maintain and benefit from the status quo.^67^ Across the globe, public sectors have been the subject of funding cuts and an increased use of private sector actors in government decision making. This critically loses a focus on achieving equity through public policy reform designed for that purpose. Our Australian case study demonstrated the impacts of this global trend and suggests that Australia could achieve better population health and equity outcomes if reforms were enacted to promote a strong public sector that invested in protecting public health and equity. It requires a sound taxation base that is critical for ensuring social and health investment, and accountable, transparent government expenditure. Many short-term outsourced projects have led to a lack of continuity in policy consideration. This leads to the loss of intellectual capital in the APS concerning ways of drafting systematic policies designed to reduce inequities.^12,58,70^

Population health outcomes are shaped not primarily by the health care system but rather by the social determinants of health. These are the conditions in which people live and work, including housing, education, income, working conditions, and discrimination.^71^ These social determinants are shaped by public policy such that policy decisions can have substantial effects on population health through affecting these drivers of health and equity outcomes, depending on how public policy affects the distribution of these social determinants of health.^72^ Our Australian case study indicates that the current use of consultancy firms to outsource government policy making carries with it a range of threats to health and equity. The negative aspects of outsourcing we identified are likely to result in public policies that foster an unfair distribution of social determinants such as housing, wealth, and income, and consequently lead to poorer health and equity outcomes. This is due to a less responsive public service, prioritizing the economy over well-being, and private over public interests, which leads to poorer outcomes for the public.

This problem was exemplified in the Robodebt disaster, in which global consulting firms were complicit and which has been deemed a catastrophic policy failure.^73^ A wide scope of negative impacts was also identified in the findings of several parliamentary inquiries into aspects of government outsourcing, including those that responded to exposés of serious misconduct in consulting firms in ways that affect good governance.^15,18,54,74^

Arguably, global consultancy firms can thus be viewed as one expression of state capture, as professional “enablers.” These also include accountants, legal advisers, public relations firms, and banks.^75^ “State capture” occurs when powerful actors abuse their power to shape the “rules of the game” in ways that benefit interest groups rather than serving the public interest.^75^ The captor can be a powerful economic actor, a conglomerate, industrial group, business interest, or part of the political elite.^76^ Multinational companies have been implicated in state capture in many countries. They lend reputational cover to domestic captor groups by providing consultancy services to plan and coordinate improper influence over different arms of the state.^76^

Unlike senior public servants, consultants are not required to implement or to be accountable for their recommended changes. These changes can be highly disruptive without offering assurance of real benefit.^39^ In the tertiary sector, expert in-house professional staff are replaced by firms that are answerable to their shareholders, not to traditional university stakeholders, including the general public.^11^

Given the range and scope of privatization, it is important to note that the debate has evolved in recent years in international financial institutions as privatization has moved toward developing economies, and due to privatization failures in the 1980s and 1990s. The context of privatization is important, with strategies linked to local conditions. Rather than the pro-privatization bias of the Washington consensus, proposals are that governments should provide better regulatory and institutional frameworks from the outset. This would include a well-functioning capital market together with employee and consumer rights.^77^

## Conclusion

There are legitimate reasons for governments to engage the services of global consulting firms in circumscribed instances when public sector capacity does not exist to deliver specific, highly specialized work. However, when outsourcing government policy-related roles to the private sector, it is critical to ensure that this work is closely supervised by capable, ethical, senior public servants to ensure probity, quality, and sound contribution to policymaking. In turn, this requires public sector capacity-building through reducing outsourcing, and by adopting “frank and fearless” advice, which will promote health and equity, and public over private interests. Unless the rampant use of consultants in policymaking is reformed, this situation will continue to have an adverse impact on population health and equity.
